# Odontoid Fracture with Accompanying Severe Atlantoaxial Instability in Elderly Patients—Analysis of Treatment, Adverse Events, and Outcome

**DOI:** 10.3390/jcm13051326

**Published:** 2024-02-26

**Authors:** Michael Kreinest, Philipp Raisch, Lukas Hörnig, Sven Y. Vetter, Paul A. Grützner, Matthias K. Jung

**Affiliations:** BG Trauma Center Ludwigshafen at the University of Heidelberg, Clinic for Trauma and Orthopedic Surgery, Ludwig-Guttmann-Straße 13, 67071 Ludwigshafen, Germanylukas.hoering@bgu-ludwigshafen.de (L.H.);

**Keywords:** odontoid fracture, atlantoaxial instability, cervical spine fracture, elderly, geriatric

## Abstract

(1) Background: In elderly patients with type II odontoid fractures, accompanying severe atlantoaxial instability (AAI) is discussed as a marker possibly warranting more aggressive surgical therapy. This study aimed to characterize adverse events as well as the radiological and functional outcomes of surgical vs. conservative therapy in patients with odontoid fracture and AAI. (2) Methods: Patients aged 65 years and older with type II odontoid fracture and AAI treated were included. AAI was assumed if the mean subluxation across both atlantoaxial facet joints in the sagittal plane was greater than 50%. Data on demographics, comorbidities, treatment, adverse events, radiological, and functional outcomes were analyzed. (3) Results: Thirty-nine patients were included. Hospitalization time was significantly shorter in conservatively treated patients compared to patients with ventral or dorsal surgery. Adverse events occurred in 11 patients (28.2%), affecting 10 surgically treated patients (35.7%), and 1 conservatively treated patient (9.1%). Moreover, 25 patients were followed-up (64.1%). One secondary dislocation occurred in the conservative group (11.1%) and three in the surgical group (18.8%). (4) Conclusions: Despite the potential for instability in this injury, conservative treatment does not seem to lead to unfavorable short-term results, less adverse events, and a shorter hospital stay and should thus be considered and discussed with patients as a treatment option, even in the presence of severe AAI.

## 1. Introduction

The odontoid fracture is the most common cervical spine injury in patients over the age of 65 [1], with a rising incidence [2,3] and considerable mortality and morbidity [4,5,6]. In the absence of clear evidence, there is no consensus on the ideal treatment for type II odontoid fractures, so the search for injury- and patient-dependent prognostic factors for treatment decisions is ongoing [7,8,9]. Since the affected patients often have serious pre-existing conditions, they require specialized conservative or surgical treatment with differentiated indications [10,11].

For this purpose, Evaniew et al. proposed a further classification of odontoid fractures based on the associated acute atlantoaxial instability (AAI) in 2015 [12]. If the mean subluxation across both atlantoaxial facet joints in the sagittal plane is greater than 50%, the AAI is classified as severe, whereas if it is less than or equal to 50%, it means the subluxation corresponds to minimal AAI. In the retrospective cohort study of Evaniew et al., patients with severe AAI showed a trend towards higher mortality and malunion rates than patients without severe AAI; thus, the importance of AAI for prognosis and treatment planning for odontoid fractures was suggested. Specifically, it was proposed that more aggressive surgical treatment might be indicated for these patients.

The number of cases of type II odontoid fractures with severe AAI specifically analyzed is low in the current literature, and mainly small case series or individual reports focusing on AAI and the rare entity of odontoid fracture with complete atlantoaxial dislocation are available [13,14,15,16,17]. Although relatively frequent, most larger studies do not look at AAI as a separate prognostic marker, and knowledge of treatment-dependent complications and outcomes is therefore limited.

However, because of the growing incidence and importance of odontoid fractures in elderly patients, more precise knowledge of the subtype of type II odontoid fractures with severe AAI is necessary, especially due to the particular severity and instability of this type of injury [12,18].

This retrospective cohort study was intended to contribute to the more precise characterization of adverse events and the outcome of this type of injury and to describe the treatment-specific advantages and disadvantages of ventral and dorsal surgery compared to conservative treatment in the affected patient collective.

## 2. Materials and Methods

The present study is a retrospective, single-center cohort study. It was conducted at a level I trauma center. The study was approved by the local ethics committee in charge (Ethics Committee of the State Medical Association Rhineland-Palatinate, Mainz, Germany). The PROCESS checklist was followed for the reporting of this study [19].

### 2.1. Patient Selection

**Figure 1:**
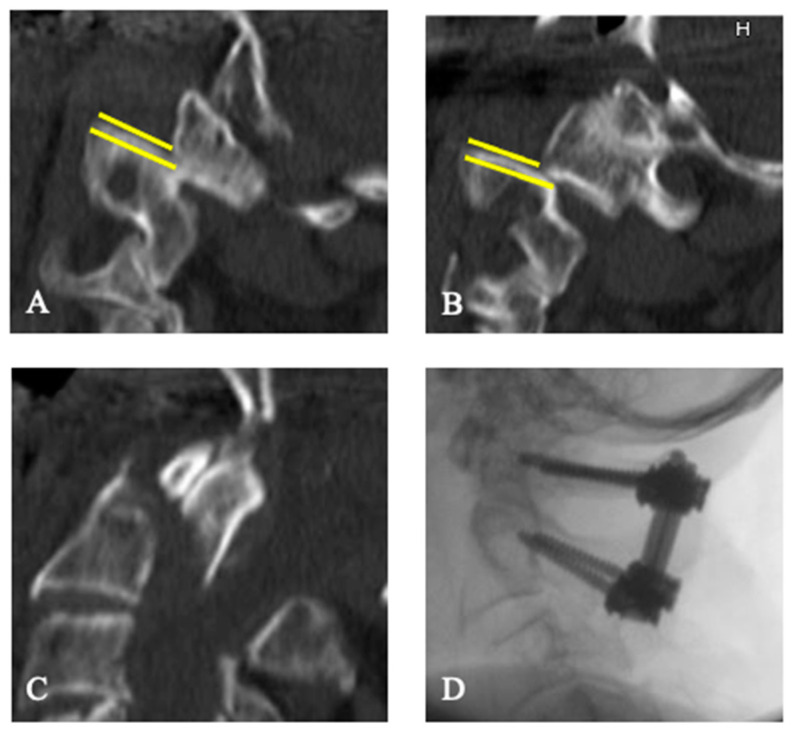
69 year old male patient with a type II odontoid fracture, according to Anderson and D’Alonzo (Case 1). Atlantoaxial instability (AAI) with a dislocation and overlap between C1 and C2 on the right ((**A**), yellow lines) and a C2-uncovering (C2U) of 85% on the left ((**B**), yellow lines) resulting in a mean C2U of more than 50%. Illustrated are the odontoid fracture (**C**) and the intraoperative fluoroscopy (**D**) after dorsal atlantoaxial instrumentation.

**Figure 2:**
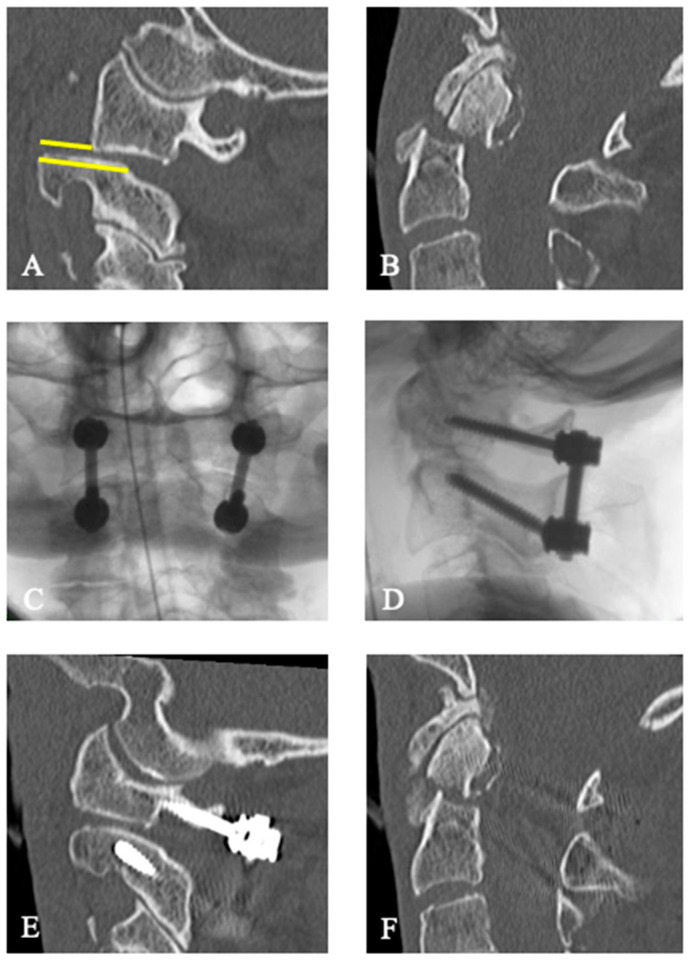
73 year old male patient with a type II odontoid fracture, according to Anderson and D’Alonzo and AAI (Case 2). The C2-uncovering is > 50% ((**A**), yellow line), and dens’ apex is dislocated (**B**). Intraoperative fluoroscopy of the dorsal atlantoaxial instrumentation in lateral (**C**) and anterio-posterior views. (**D**). Postoperative computed tomography with the correct position of the lateral C1/C2 joint (**E**) and reposition of the dens’ apex (**F**).

All patients who met the following inclusion criteria were included: (1) Patients treated for odontoid fracture type II according to Anderson and D’Alonzo [20] from January 2012 to December 2017, (2) age 65 years and older, and (3) severe AAI with a mean C2-uncovering (C2U) of at least 50% according to Evaniew et al. [12]. The C2U is defined as the largest exposed articular surface of the cranial joint of the axis on the right and left parasagittal computerized tomography (CT) reconstruction scans (Figure 1 and Figure 2, yellow lines) [12]. While the original publication on AAI does not elaborate on this differentiation, we chose to only include patients with instability resulting in subluxation of the articular surfaces in the same direction (i.e., anteriorly or posteriorly).

For each patient, AAI was evaluated using a CT scan. The C2U in all CT scans was independently evaluated by two spine surgeons; disagreements were resolved via consensus. All measurements were performed with the IMPAX software (Version 6.5.5.1608; AGFA Healthcare; Mortsel, Belgien). Injuries were also classified according to Grauer [21] and the AO Spine Upper Cervical Injury Classification System [22]. Patients with pathological fractures of the dens and patients with rheumatoid arthritis were excluded from the study since an increased risk of AAI has been described in these conditions [23,24].

### 2.2. Patients’ Data

Demographic data as well as prehospital and treatment data and adverse events (AEs) of every patient are documented in the hospital database as a standard procedure. The following parameters were collected for all patients: age, sex, Glasgow Coma Scale (GCS) upon admission, concomitant injuries, neurological function, and severity of AAI.

### 2.3. Treatment of the Patients

The patients in the present study were treated conservatively or surgically according to the preference of the treating spine surgeon and the patients. Apart from injury morphology, patient condition and comorbidities were considered.

Conservative treatment was performed by immobilizing the cervical spine in a rigid cervical collar for six weeks. All conservatively treated patients underwent outpatient clinical examinations and radiological evaluations with CT scans of the cervical spine.

**Figure 3:**
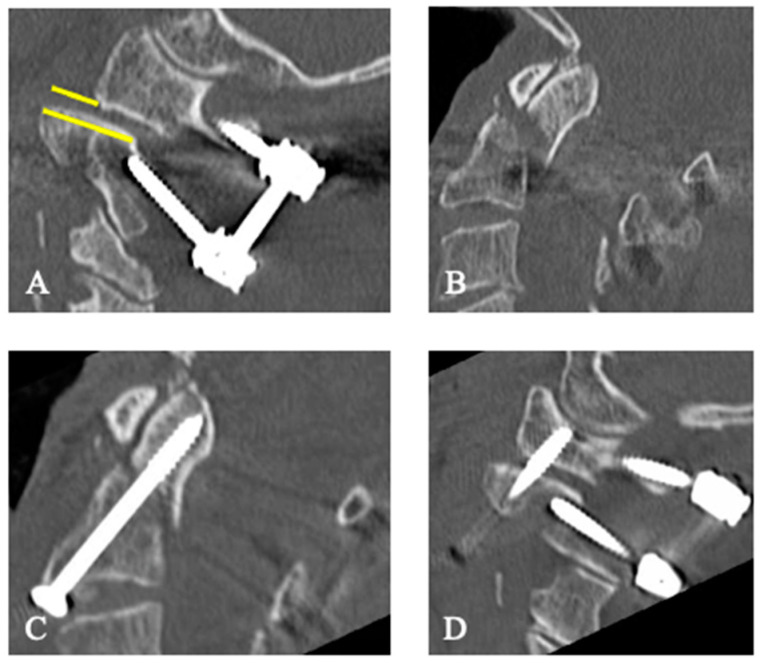
After dorsal surgical stabilization of Case 1 (Figure 1), there was a secondary dislocation with C2-uncovering ((**A**), yellow line) with dislocation of the odontoid (**B**). Ventral screw osteosynthesis of the odontoid (**C**) and additional fusion of C1 and C2 were performed (**D**). The postoperative CT scan shows the correct position in the C1/C2 joint (**D**).

Two different surgical methods for stabilizing odontoid fractures were performed. Ventral screw osteosynthesis was performed using a standard Smith–Robinson approach to the upper cervical spine [25]. Two guide wires were inserted into the odontoid from the ventrocaudal. Screws were inserted over the guide wires (Figure 3). Dorsal instrumentation was performed via a median posterior approach, according to the surgical technique of Harms et al. [26].

### 2.4. Evaluation of Adverse Events

Surgical adverse events (SAE) were evaluated according to the classification of Dindo et al. [27]. Grade I is defined as complications that do not require treatment. Grade II is defined as complications that require pharmacological intervention only. Grade III is defined as complications that require surgical intervention, and grade IV includes all complications that are life–threatening. The death of patients is classified as grade V. If patients had multiple SAEs, they were classified according to the complication with the highest grade. Non-surgical adverse events (NAEs) were also assessed and graded.

### 2.5. Clinical Evaluation

If possible, patients were examined for outpatient follow-up in the study clinic. At the final follow-up, the range of motion (ROM, degrees) of the cervical spine was analyzed with a goniometer. Flexion and extension, lateral bending to the right and left, and rotation to the right and left were measured. All patients were questioned about pain and underwent examination for neurological deficits.

### 2.6. Radiological Evaluation

A CT scan of the cervical spine was performed on admission to the hospital and postoperatively. In addition, further CT scans were performed on the follow-up to evaluate fracture position and detect any secondary dislocation (yes/no).

### 2.7. Endpoints

The number and type of AEs during the initial hospital stay were determined, as described above. The primary endpoints at the outpatient follow-up were functional outcome with ROM of the cervical spine and secondary dislocation. Patients who could not be followed-up after discharge were not included in this analysis. Furthermore, operation time (minutes), time at the hospital (days), and the presence of neck pain and neurological deficits at follow-up were determined.

### 2.8. Statistical Analysis

The normal distribution of the data was assessed using the Shapiro–Wilk test. Normally distributed, continuous variables were described in means and ranges. Skewed, continuous variables were described with a median and a confidence interval (CI). Parametric, continuous data were analyzed using a two-sided independent sample *t*-test or the ordinary one-way analysis of variance (ANOVA). The Mann–Whitney U test, or Kruskal–Wallis test, was used to analyze the non-parametric data. Distributions of categorical variables were assessed using a Chi-squared test. To assess statistical significance, two-tailed *p*-values were calculated, and statistical significance was assumed for *p* < 0.05. The statistical evaluation was performed using Prism (GraphPad, version 8.2.1, San Diego, CA, USA).

## 3. Results

### 3.1. Patient Characteristics

Thirty-nine patients were included in the study. The mean age was 78.4 years (range: 65–93 years, Table 1). The pre-existing conditions and concomitant diseases are presented in Table 1. Between the treatment groups, there were no significant differences between age, gender, and Grauer classification distributions [21] (*p* = 0.0998), as also shown in Table 1. Due to the inclusion criteria, all injuries were classified as C2 Type C, M2, in the AO Spine Upper Cervical Injury Classification System [22].

Preoperatively, injury-related neurological abnormalities were present in one patient from the ventral surgery group (central cord syndrome) and three patients from the dorsal surgery group (two with central cord syndrome and one patient with Brown-Séquard syndrome), all classified as N3, according to AO Spine. No patient presented with a complete spinal cord injury.

### 3.2. Treatment

Eleven patients were treated conservatively (28.2%, Table 2). A ventral surgical approach was used in eight patients (20.5%), and 20 patients (51.3%) underwent surgery with dorsal instrumentation. The mean operation time of patients with ventral surgery was significantly shorter than that of patients with dorsal surgery (52 vs. 176 min, *p* < 0.0001). Hospitalization time was significantly different in the three treatment groups (*p* < 0.0001): conservatively treated patients had the shortest hospitalization time (median 5 days), followed by patients with ventral surgery (median 11 days) and dorsal surgery (median 14.5 days) (Table 2).

### 3.3. Adverse Events

Adverse events occurred in 11 patients (28.2%), affecting 10 surgically treated patients (35.7%) and 1 (9.1%) conservatively treated patient during the hospital stay. SAEs occurred in 5 of the 28 surgically treated patients (17.9%), and NAEs occurred in 6 patients in the total sample (15.4%), 1 from the conservative (9.1%), and 5 from the surgical group (18.8%). The dorsal surgery group was more frequently affected by SAEs than the ventral surgery group (dorsal surgery group *n* = 4, 20%; ventral surgery group *n* = 1, 12.5%). Ventral surgery had to be revised once because of a secondary dislocation (grade III). Dorsal instrumentation had to be revised once because of secondary dislocation (Figure 1 and Figure 3), and one patient had to be revised because of acute epidural hematoma. Both cases were classified as grade IV because of the danger of life-threatening spinal cord compression. One patient in the dorsal surgery group underwent revision surgery for non-life-threatening epidural hematoma (grade III), and one patient developed dry wound necrosis without need for intervention (grade I). No neurological deterioration occurred during the hospital stay.

Representing NAEs, delirium (grade II) occurred in one patient in the conservative group (9.1%) and in two patients in the dorsal surgery group (10.0%). One patient from the dorsal surgery group (5.0%) had to be treated for hypokalemia (grade II). Two patients from the dorsal surgery group (10.0%) suffered urosepsis (grade IV).

### 3.4. Outcome

A total of 25 patients could be followed up radiologically and clinically (follow-up rate: 64.1%, Table 3). The follow-up could be performed after an average of 5.2 months (range: 1–12). From the conservative patient group, 9 patients (81.8%); from the ventral surgery group, 5 patients (62.5%); and from the dorsal surgery group, 12 patients could be reexamined (55.0%). Between the follow-up groups, there were no significant differences between age and gender distributions. The mean follow-up times in the treatment groups were comparable, with 5.3 months in the conservative group, 4.8 months in the dorsal surgery group, and 6.0 months in the ventral surgery group (*p* = 0.4107).

#### 3.4.1. Functional Outcome

Cervical spine ROM was measured in all patients at follow-up (Table 3). Between the treatment groups, there was no significant difference in flexion (*p* = 0.7863) and extension (*p* = 0.8743), in lateral bending to the right (*p* = 0.1452) and left (*p* = 0.3191), or in rotation to the right (*p* = 0.8565) and left (*p* = 0.4966). Cervical spine ROM was markedly reduced across all treatment groups compared to the physiological state. At follow-up, nine patients reported pain (36.0%, Table 3). Only one patient from the conservative group (11.1%) reported pain, compared to eight patients from the surgical groups (50.0%), of which three patients were from the ventral surgery group (60.0%) and five patients from the dorsal surgery group (45.5%).

Neurologic abnormalities were present in four patients (16.0%, Table 3). There were two patients with an unsteady gait (conservative treatment and dorsal surgery group). The other two patients reported continued brachialgia after dorsal instrumentation.

#### 3.4.2. Secondary Dislocations

There were four secondary dislocations of the odontoid fracture (16.0%, Table 3). One dislocation occurred in the conservative group (11.1%), three in the surgical group (18.8%), two in the ventral surgery group, and one in the dorsal surgery group. One patient from the dorsal surgery group underwent revision because of the dislocation (Figure 3).

## 4. Discussion

The present study describes complications as well as the functional and radiological outcomes of surgical and conservative treatment in 39 elderly patients with type II odontoid fractures with severe AAI. To the authors’ knowledge, since Evaniew et al. suggested AAI as a prognostic variable possibly directing treatment decisions in odontoid fractures in 2015 [12], no larger-scale systematic investigation of this concept has been pursued.

Regarding functional outcome, there were no significant differences in ROM between treatment groups, and conservatively treated patients had pain less frequently. Secondary dislocation rates were lower than in surgically treated patients. The hospital stay was significantly shorter with conservative treatment. Finally, there was a higher rate of AEs in the surgically treated cohort than in the conservative group. Accordingly, our results suggest that, even in the presence of severe AAI, surgical treatment can be safely postponed, if necessary, without concern for higher rates of AE’s or secondary dislocations. Conservative treatment should be discussed as a viable treatment option.

### 4.1. Evidence in Treatment of Type II Odontoid Fractures

Generating scientific evidence for treatment decisions in elderly patients with type II odontoid fractures has been of paramount interest in recent years. Some high-quality large-scale retrospective and prospective studies, as well as systematic reviews and meta-analyses, have investigated treatment options and outcomes showing lower rates of mortality in surgically treated patients [3,4,5,7,28,29], even after adjustment for comorbidities [4], as well as improved functional outcomes in patients treated surgically [5]. One recent prospective multicenter study, on the other hand, demonstrated a very good functional outcome with conservative therapy [30].

However, these studies did not stratify patients according to accompanying AAI and thus are not suitable to determine the possible effect of severe AAI on complications and outcomes or whether surgical or conservative treatment leads to better results.

### 4.2. Evidence in Treatment of Odontoid Fractures with AAI

The literature investigating the treatment of odontoid fractures with accompanying atlantoaxial instability, subluxation, or dislocation is mainly composed of case reports or smaller case series of patients of various ages [13,14,15,16,17]. One issue in these reports is that, frequently, there is no clear definition of AAI, or differing definitions do not permit direct comparisons of injuries and treatment results. Reports on conservative treatment are underrepresented.

Wang et al. [13] published a case series of 12 patients with odontoid fracture and instability of adjacent segments, as well as 5 patients with atlantoaxial subluxation. All were treated surgically with favorable results, i.e., pain relief, odontoid fracture union within six months, and improved neurological deficits. There were no surgical complications. Of note, all these patients underwent treatment with a cervical brace for three months after surgery. Wang’s AAI patients were aged 45 to 60 years, thus being much younger than the cohort in this study, possibly contributing to the absence of complications. There was no conservative treatment group, and the long postoperative treatment in a cervical orthosis of three months leaves one to question whether this strict regime of immobilization alone might have led to stable healing.

Recent case reports describe the management and outcome of patients with the rare combination of odontoid fracture and complete atlantoaxial dislocation [14,15,16,17]. All the cited case reports opted for surgical fixation after reduction and reported favorable results. The literature shows that the entity of the odontoid fracture with complete atlantoaxial dislocation is rare, possibly because of the high chance of severe spinal cord injury resulting in the immediate death of the affected individual.

In contrast, Evaniew’s cohort [12] demonstrated a high prevalence of severe AAI accompanying odontoid fractures in elderly patients of 14%, underlining the general importance of better understanding this injury of the upper cervical spine.

### 4.3. Adverse Events and Mortality

We observed AEs in 9.1% of conservatively treated patients and 35.7% of surgically treated patients. In contrast, Vaccaro et al.’s prospective study of 159 elderly patients with odontoid fractures reported AEs in 36% of conservatively treated patients and 30% of surgically treated patients [5]. Thus, we observed a much lower rate of AEs in conservatively treated patients, both compared to our surgical cohort as well as to Vaccaro’s surgical and conservative cohort. It must be stressed, though, that our retrospective design is inferior to a prospective design when analyzing AEs.

When comparing surgical approaches, we saw higher rates of SAEs in the dorsal group, which is in accordance with large-scale studies [7,31]. We also saw significantly longer surgical times for dorsal surgery, possibly contributing to the higher rate of AEs in this group.

One concern when treating patients with AAI, in particular conservatively, is neurological deterioration due to instability and resulting spinal cord compression [12]. We did not witness neurological deterioration in any of the treatment groups, even in cases with secondary dislocation. In fact, spinal cord injury in geriatric odontoid fractures is rare, possibly because of the relatively wide spinal canal at this level [32]. This seems to be equally true in cases accompanied by severe AAI.

In our cohort, patients treated surgically spent more than twice as long in the hospital as patients treated conservatively. This, in our eyes, illustrates a weighty advantage of conservative treatment, as hospitalization is often associated with prolonged bed rest, which in turn can lead to potential harm in the form of infections and sarcopenia [33].

Finally, while mortality was not analyzed in our study, Evaniew et al. noted a significantly higher rate of mortality in patients with severe AAI of 35% vs. 14% in patients without severe AAI but could not demonstrate a statistically significant positive effect of surgical treatment on survival in patients with severe AAI [12].

The presence of severe AAI in geriatric patients with odontoid fractures might lead treating surgeons to indicate surgical treatment earlier and more liberally because of fear of dislocation, neurological injury, and other AEs. Our data, in contrast, suggest that surgical treatment can be delayed in the first weeks and months after the injury, with low rates of secondary dislocation and other AEs. Postponing surgery might be necessary due to patient factors such as comorbidities needing optimization before surgery or patients being skeptical about surgery. Favorable results with lower AE rates might be possible with conservative treatment.

To make a definitive statement, however, further research is needed concerning cross-over rates from conservative to surgical treatment due to mid- and long-term problems and concerning outcomes in patients with early vs. delayed surgery.

### 4.4. Functional Outcome

Functional outcome with ROM was evaluated at follow-up. In most studies comparing the different treatment methods, patients with dorsal instrumentation show significantly impaired movement, especially in rotation, when compared to patients with ventral screw osteosynthesis, where no motion segment is stiffened [34,35]. In our cohort, there was no significant difference in ROM between the different treatment groups at follow-up, with markedly reduced ROM even in patients with conservative treatment or ventral screw osteosynthesis. This might be a result of post-treatment with a cervical collar. However, we observed a much lower prevalence of pain at follow-up in patients treated conservatively.

### 4.5. Radiological Outcome

Evaniew reported nonunion in 29% of patients with severe AAI regardless of treatment modality, as opposed to 10% nonunion in patients without severe AAI [12], which was statistically significant. However, mean follow-up in the cited study was only 4.4 months; minimum and maximum follow-up were not reported. It is thus unclear whether confident statements on the status of union can truly be derived. With our follow-up rate of 64.1% and a mean duration of 5.2 months, we were unable to do so.

When analyzing radiological nonunion as an outcome parameter, it is important to consider that nonunion of the odontoid might not negatively affect function and quality of life [24,30,34,36]. Generally, nonunion rates of odontoid fractures in the geriatric population are reported heterogeneously in the literature [7], complicating comparisons. A systematic review of 640 cases reported nonunion in 39% of patients treated conservatively in a collar and in 27% and 11% of patients treated surgically with anterior and posterior fusion, respectively [3]. A large prospective multicenter study by Chibarro et al. [30], including 260 elderly patients with type II odontoid fractures mostly treated conservatively, reported excellent functional outcomes even in the 50% of patients without bony healing.

### 4.6. Limitations

The present study is limited by its single-center, retrospective design. The decision on individual treatment was made by the treating surgeon and the patient based on suspected instability, comorbidities, patient condition, and patient preference. Thus, as a major weakness of this retrospective study, systematic errors about patient therapy and selection bias cannot be excluded.

The total patient number might be too small to detect significant differences in patient outcomes between the treatment groups. The number of followed-up patients is limited (follow-up rate of 64.1%) and might have introduced bias toward survival and favorable outcomes. Mortality, an endpoint of high interest in other studies, was not analyzed. The mean follow-up time of 5.2 months is not sufficient to obtain reliable data on long-term problems like painful nonunions. Vaccaro et al. [5] reported a high crossover rate from conservative to surgical treatment in odontoid fractures because of this long-term problem, while Chibbaro et al. [26] reported low crossover rates. ROM was measured with standardized goniometers but by different orthopedic surgeons, possibly limiting comparability between patients.

Finally, AAI was evaluated and stratified according to Evaniew et al. [12] based on static post-injury CT scans. It must be assumed that this examination is of limited sensitivity for the detection of severe AAI, as it misses patients with instability but a relative reduction on the CT table.

## 5. Conclusions

Despite the high potential for instability in this injury, nonsurgical treatment does not seem to lead to unfavorable early results. Conservative treatment leads to fewer adverse events and a shorter hospital stay in the short term. It therefore seems safe to delay surgery if necessary, and conservative treatment should be considered and discussed with patients as a treatment option, even in the presence of severe AAI.

## Figures and Tables

**Table 1:** Characterization of patients in the different treatment groups.

	Conservative Treatment *n* = 11	Ventral Surgery *n* = 8	Dorsal Surgery *n* = 20	*p*
Mean age, years (range)	82.6 (66–93)	79.9 (67–90)	78.8 (65–92)	0.5049
Classification according to Grauer, *n* (%)				0.0998
Grauer 2 A	2 (18.2)	0 (0.0)	0 (0.0)	
Grauer 2 B	8 (72.7)	5 (62.5)	17 (85.0)	
Grauer 2 C	1 (9.1)	3 (37.5)	3 (15.0)	
Sex, *n* (%)				0.5788
Female	5 (45.5)	5 (62.5)	8 (40)	
Male	6 (54.5)	3 (37.5)	12 (60)	
Median GCS (CI)	15 (14–15)	15 (14–15)	15 (15–15)	0.1448
Medical comorbidities, *n* (%)				
Cardiological	7 (63.6)	5 (62.5)	14 (70.0)	
Pulmonary	3 (27.3)	1 (12.5)	1 (5.0)	
Metabolic	6 (54.5)	3 (37.5)	9 (45.0)	
Traumatic	9 (81.8)	3 (37.5)	6 (30.0)	
Neurological	0 (0.0)	2 (25.0)	6 (30.0)	
Psychiatrically	3 (27.3)	1 (12.5)	2 (10.0)	
Allergies	0 (0.0)	0 (0.0)	2 (10.0)	
Cancer	0 (0.0)	0 (0.0)	2 (10.0)	
Other	3 (27.3)	2 (25.0)	3 (15.0)	
Concomitant spine injuries, *n* (%)				
Upper cervical spine	6 (54.5)	4 (50.0)	6 (30.0)	
Lower cervical spine	0 (0.0)	0 (0.0)	0 (0.0)	
Spinal column	0 (0.0)	1 (12.5)	0 (0.0)	
Concomitant injuries, *n* (%)				
Traumatic brain injury	5 (54.5)	0 (0.0)	2 (10.0)	
Pelvic fracture	0 (0.0)	1 (12.5)	0 (0.0)	
Fracture of the extremities	2 (18.2)	2 (25.0)	3 (15.0)	
Skull fracture	6 (54.5)	2 (25.0)	4 (20.0)	
Traumatic neurological deficits	0 (0.0)	1 (12.5)	3 (15.0)	

CI, confidence interval; GCS, Glasgow Coma Scale.

**Table 2:** Operation time, hospital stay, and adverse events corresponding to the different treatments.

Treatment, Complications	Conservative Treatment *n* = 11	Ventral Surgery *n* = 8	Dorsal Surgery *n* = 20	*p*
Mean operation time, minutes (range)		52.3 (23–85)	176.2 (86–315)	<0.0001
Median time at the hospital, days (CI)	5.0 (3–10)	11.0 (5–20)	14.5 (13–21)	<0.0001
Surgical adverse events, *n* (%)				
Grade I	0 (0.0)	0 (0.0)	1 (5.0)	
Grade II	0 (0.0)	0 (0.0)	0 (0.0)	
Grade III	0 (0.0)	1 (12.5)	1 (5.0)	
Grade IV	0 (0.0)	0 (0.0)	2 (10.0)	
Grade V	0 (0.0)	0 (0.0)	0 (0.0)	
Non-surgical adverse events, *n* (%)				
Grade I	0 (0.0)	0 (0.0)	0 (0.0)	
Grade II	1 (9.1)	0 (0.0)	3 (15.0)	
Grade III	0 (0.0)	0 (0.0)	0 (0.0)	
Grade IV	0 (0.0)	0 (0.0)	2 (10.0)	
Grade V	0 (0.0)	0 (0.0)	0 (0.0)	

**Table 3:** Characterization of patients’ functional and radiological outcomes, corresponding to different treatments.

Functional Outcome	Conservative Treatment *n* = 9	Ventral Surgery *n* = 5	Dorsal Surgery *n* = 11	*p*
Mean ROM,				
degree (range)				
Flexion	26.0 (20–30)	24.0 (20–30)	25.0 (20–30)	0.7863
Extension	23.3 (10–40)	22.0 (10–40)	26.0 (10–40)	0.8743
Lateral bending right	23.1 (10–35)	23.0 (10–35)	16.4 (10–30)	0.1452
Lateral bending left	23.8 (15–35)	23.0 (10–35)	18.6 (10–30)	0.3191
Rotation right	33.1 (10–45)	35.0 (10–60)	37.7 (10–60)	0.8565
Rotation left	30.6 (15–40)	33.0 (10–60)	38.6 (15–60)	0.4966
Pain, *n* (%)				
Yes	1 (11.1)	3 (60.0)	5 (45.5)	
No	8 (88.9)	2 (40.0)	6 (54.5)	
Neurology, *n* (%)				
Yes	1 (11.1)	0 (0.0)	3 (27.3)	
No	8 (88.9)	5 (100.0)	8 (72.7)	
**Radiological Outcome**	**Conservative Treatment** ***n* = 9**	**Ventral Surgery** ***n* = 5**	**Dorsal Surgery** ***n* = 11**	
Dislocation, *n* (%)				
Yes	1 (11.1)	2 (40.0)	1 (9.1)	
No	8 (88.9)	3 (60.0)	10 (90.9)	

## Data Availability

All data and materials regarding this study are available from the corresponding author on reasonable request.
